# The impact of anatomical recipient liver abnormalities on rat liver transplantation

**DOI:** 10.3389/fvets.2025.1593434

**Published:** 2025-07-15

**Authors:** Yongfeng Chen, Wenzhong Li, Guoyong Chen, Shaotang Zhou

**Affiliations:** ^1^6th Hepatopancreaticobiliary Surgery of Henan Provincial People’s Hospital, People’s Hospital of Zhengzhou University, Zhengzhou, China; ^2^Acupuncture and Massage Department, Henan University of Chinese Medicine, Zhengzhou, China

**Keywords:** orthotopic liver transplantation, liver deformity, outcome, two liver outflows, liver accessory lobe

## Abstract

**Background:**

Orthotopic rat liver transplantation (OLT) is widely used in basic research; normal liver anatomy and structures are attributable to its success, but its deformities are complicated by a negative OLT.

**Methods:**

For tolerance induction, we used OLT from Lewis to Brown Norway (BN) rats as a chronic rejection model and encountered two anatomical deformities in the recipient livers. The outcomes of OLT were analyzed.

**Results:**

Of the 47 liver transplantations, the accessory liver lobe occurred in four cases, and bifurcations of liver outflow occurred in five cases in BN rats. For the accessory liver lobe, we discontinued OLT in one patient with a large accessory liver lobe. Two rats died from pneumothorax upon separation, and one case succeeded with a small lobe. For two vein outflow orifices of the liver, we succeeded in OLT due to its reconstruction in one case; however, the recipient died 1 week later in one case, after one small orifice was sutured. We failed in three cases due to thrombosis following OLT. Among the 38 rats with normal liver anatomy, only four failed to survive the LT. There were significant differences in OLT success (*p* < 0.01).

**Conclusion:**

The recipient’s liver abnormal anatomical structure has a negative impact on OLT, suggesting that pretransplant comprehensive screening is important and that clinicians are cautious in clinical practice.

## Introduction

Rat OLT has received wide acceptance as a small animal model in research on ischemia-reperfusion injury, immunology, and transplant medicine, especially tolerance induction, since the cuff technique was developed by Lee et al. (1, 2). OLT remains a complicated procedure in which normal liver anatomy is a prerequisite for its success. In this scenario, OLT comprises four or five anastomoses or reconnections of the suprahepatic inferior vena cava (SHVC), portal vein, infrahepatic inferior vena cava (IHVC), bile duct, and/or liver artery. Any anatomic abnormality of the recipient liver complicates this surgery and possibly leads to failure (3–6). Here, we first report the anatomical deformities of the recipient liver that have a negative impact on OLT.

## Methods

We have been conducting a project on liver regeneration and immunological tolerance using stem cells (a detailed protocol out of scope here) for our study. We have also performed rat OLT from Lewis to BN, which is well recognized as a chronic rejection model (5). Male Lewis rats (weighing 200–280 g) and BN served as donors and recipients, respectively, and were purchased from Vital River Laboratory Animal Technology Corporation. The animals were housed and cared for in a standard environment with a 12-h cycle of light and darkness at a controlled temperature. They were allowed free access to standard food and water. Each rat was fasted for 12 h prior to OLT. In the case of fatal intraoperative events, we discontinued surgery and immediately euthanized the rats such that the heartbeat gradually stopped upon the maximal inhalation of isoflurane. All experiments were approved by the Ethics Committee of Henan Integrated Traditional Chinese and Western Medicine Hospital and conducted in compliance with the standards for animal use and care set by the ARRIVE guidelines and the Institutional Animal Care Committee (No. HNTCMDW-20240829).

### Surgical procedure

Some modifications were made for isoflurane inhalation anesthesia. During the donor procedure, one microsurgeon made a transverse incision to enter the abdominal cavity of the rat. They first flushed the donor liver through the aorta with heparinized normal saline (50 U/mL) and then reflushed through the portal vein (PV) with 4°C lactated Ringer’s solution. The artery branch segment from the celiac artery to the proper liver artery was retained, and the splenic artery and the left gastric artery were ligated. The graft was explanted and stored in lactated Ringer’s solution. Cuffs were prepared for the PV (outer diameter 2.6 mm, inner diameter 2.2 mm) and the infrahepatic vena cava (IVC). The outer diameter was 3.2 mm, and the inner diameter was 2.8 mm. The cold storage time was less than 3 h in all cases. In the recipient, a midline incision was made, and the surgical field was exposed using three homemade retractors with the clip. All the ligaments of the liver were separated with an electric bipolar. The proper liver artery was ligated. Next, the surgeon made a blunt separation behind the liver to create a tunnel. The recipient PV and IVC were sequentially clamped with microvascular clamps, and isoflurane was immediately decreased to 0.2%. Through the tunnel, mosquito forceps were positioned on the part of the diaphragm ring (left side) to occlude the suprahepatic vena cava (SHVC), and the native liver was removed. The SHVC was anastomosed with an 8–0 polypropylene suture. The mosquito forceps were displaced with a vascular bulldog on the real SHVC while the diaphragm ring was declamped. The PV was reconnected with the cuff. Blood flow was restored when the clamp on the PV was released, and the an-hepatic time was generally less than 20 min. IVC reconnection was performed using the same methodology as was used for the PV. The microsurgeon ligated the gastroduodenal artery proximally and opened the common hepatic artery, into which the stent (made in-house) in the donor hepatic artery was inserted and secured (5). Bile duct continuity was achieved when a tube (0.8 mm in outer diameter) in the donor bile duct was inserted into the recipient bile duct. For the small accessory liver lobe, which extended into the thorax, we dissected far from the diaphragm to avoid pneumothorax. The large accessory liver lobe was difficult to resect, and the residual lobe compromised respiration. The two orifices of the vein outflow were reconstructed into one orifice. The abdomen was closed with two layers, and the incisional wound was coated with lidocaine cream. The animals were placed in a cage under infrared light. Both 10% glucose solution and purified water were supplied for 3 days, after which regular food and tap water were provided. OLT in which the recipient survived for 2 days was considered successful.

### Statistical analysis

The success rates of LT in the different groups were evaluated with the *X*^2^ test, which was calculated artificially, and a *p*-value of <0.05 was considered significant.

## Results

**Figure 1:**
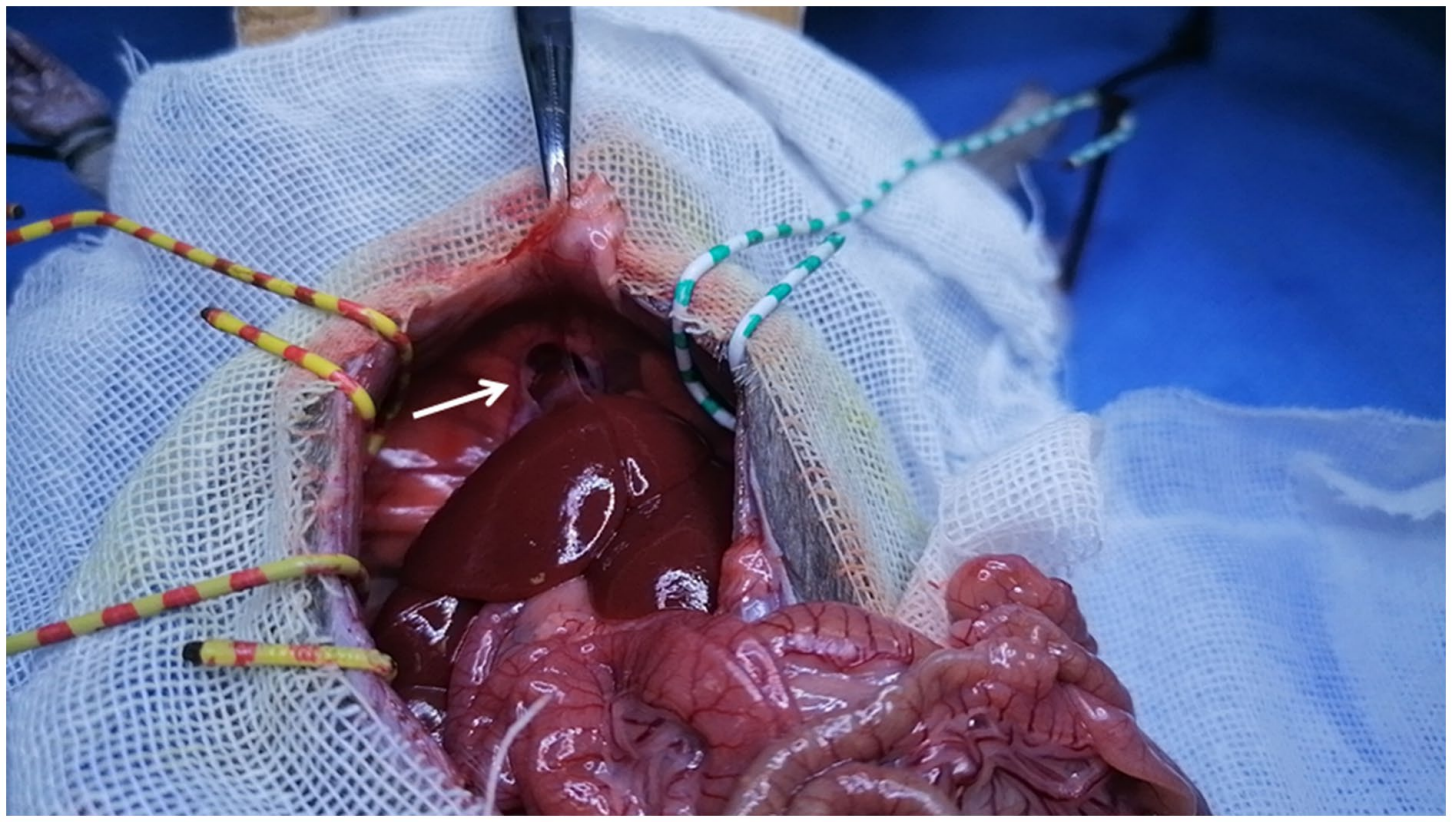
Accessory liver lobe. It is connected to the diaphragm (arrow).

**Figure 2:**
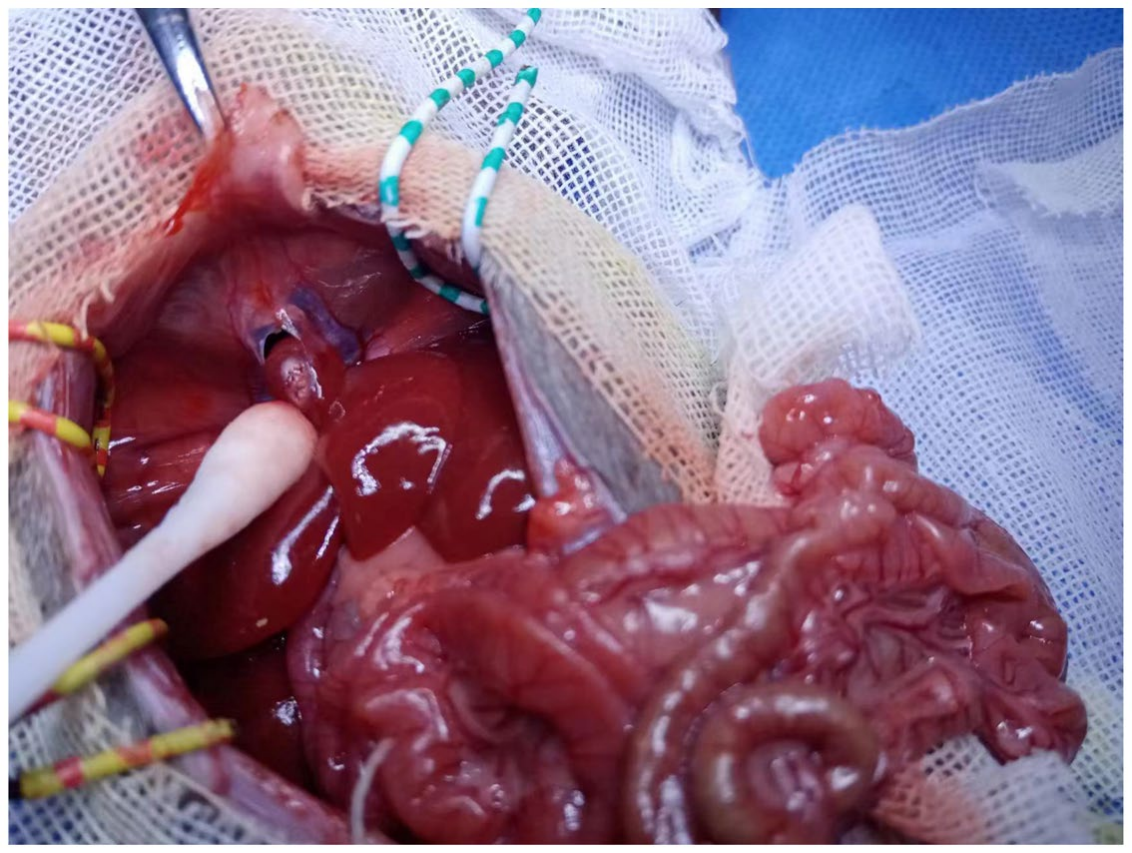
Accessory liver lobe. Pneumothorax occurred and caused death after separation.

**Figure 3:**
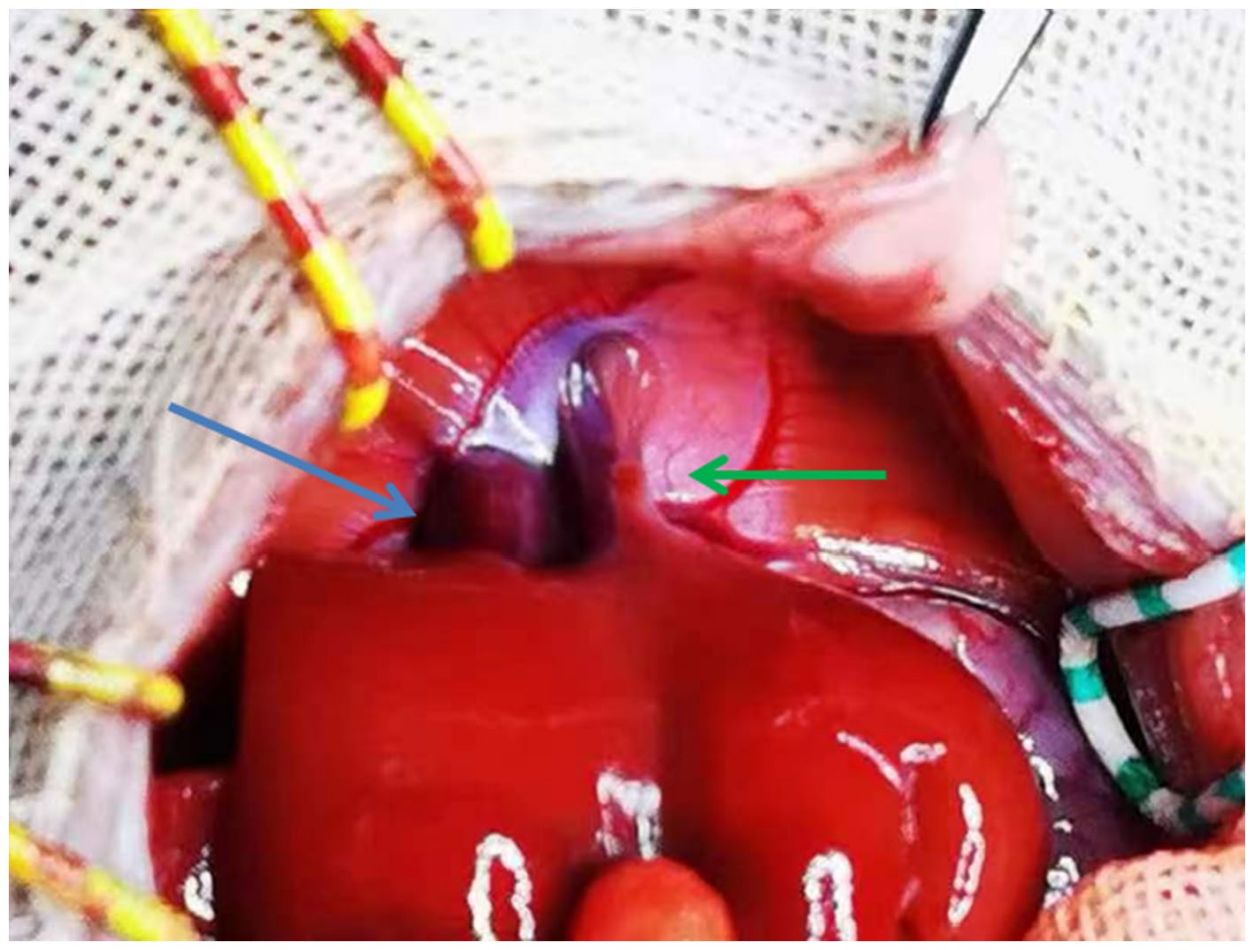
Two outflows in the recipient rat liver (arrows, green, and blue).

**Figure 4:**
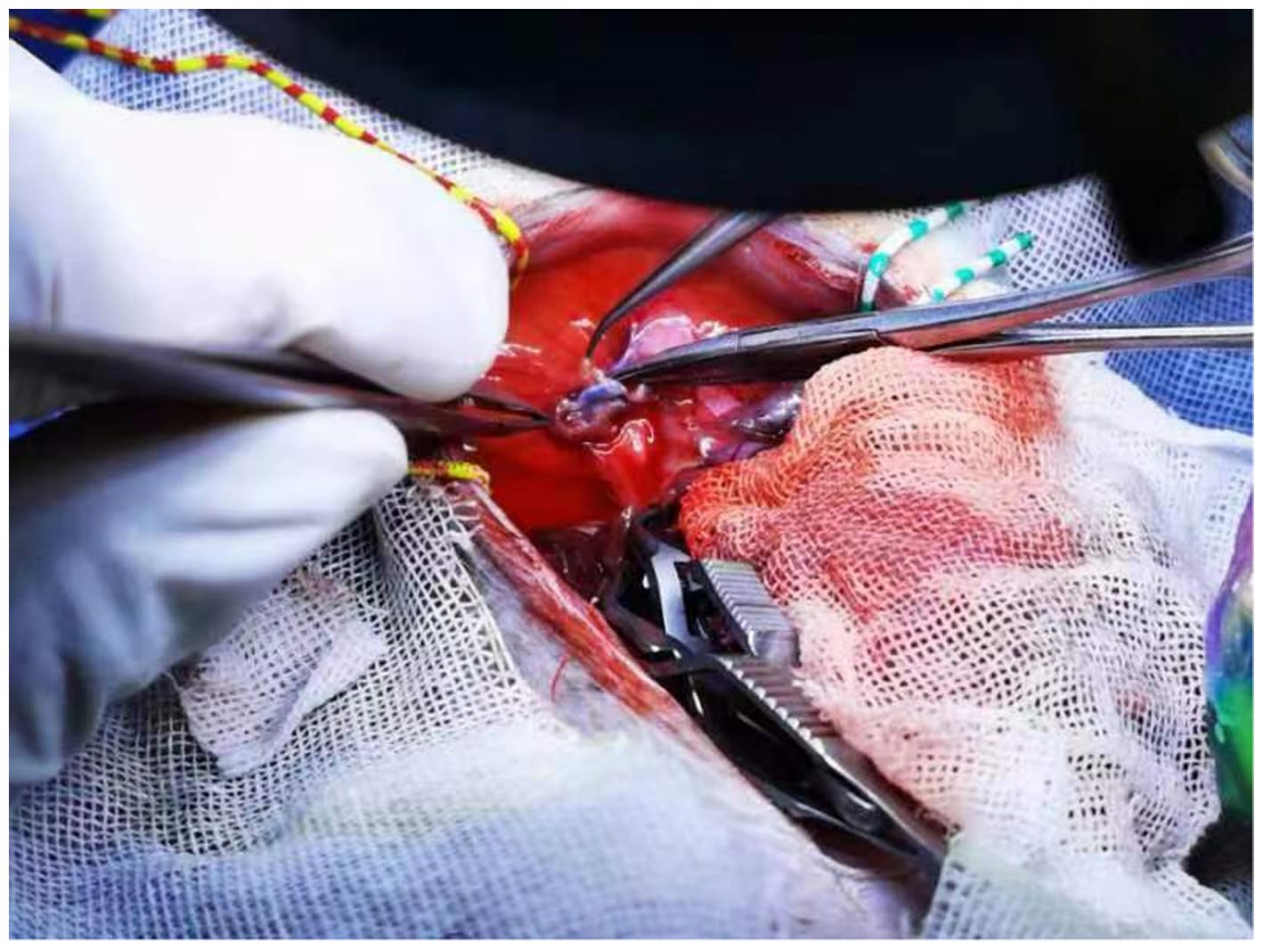
Two outflows of the liver from the recipient rat. The small orifice was closed.

For explants of the recipient liver, we encountered two anatomical abnormalities in BN rats: an accessory liver lobe in four cases (4/47) and two liver outflows in five cases (5/47) (Table 1). The accessory liver lobe was surrounded by a ligament connecting the diaphragm and extended to cause Morgagni hernia (Figures 1, 2). One was the small accessory lobe, which was separated from the main liver using the bipolar cauterizer, and OLT was successful (Supplementary Video 1). The accessory lobes were larger in three cases. Separation was unsuccessful due to pneumothorax and led to death in two cases, and OLT was stopped directly in one case. Abnormal outflow orifices of the liver occurred in five cases (Figure 3). Two orifices became septic due to the muscular diaphragm and converged into one inferior vena cava in the thoracic cavity. OLT was completed due to reconstruction of the outflows in one case, and the host died within 1 week. OLT succeeded in one case after one small orifice (left) was sutured (Figure 4). OLT failed in three cases because of large thrombi shortly upon systemic blood restoration after two orifices were reconstructed into one. Among the remaining 38 rats, 34 underwent OLT successfully, whereas four failed due to blood loss associated with missed suturing. There were significant differences in LT success (*p* < 0.01).

**Table 1:** LTs with deformities.

Liver	Cases	LT success	LT failure
Accessory lobe	4	1	3
Outflows	5	1	4
Normal	38	34	4

*X*^2^ = 18.386, *p* < 0.01.

## Discussion

OLT has become a well-established and effective treatment exclusively for end-stage liver diseases that benefits many patients. LT is a complicated surgery that requires additional basic studies. Small animal liver transplantation models, such as rats, have important value, and rats hold a prominent position in the field of OLT, especially in chronic rejection studies (3–6).

Surgically, LT is a challenging procedure involving the dissection and removal of a diseased liver. With subsequent implantation of the graft, effective reconstruction of vascular and biliary anastomoses, including potent outflow, is fundamentally necessary. This requires good conditions and normal anatomy of the host, including cardiac, pulmonary, and renal functionalities (1, 2). The accessory liver lobe (ALL) is a congenital ectopic liver tissue that is affected by the embryonic dysplasia described in 1767 (7, 8). Two types of ALLs are found: an accessory lobe joining normal liver tissue and another part that is completely separated by the diaphragm and often extends into the thoracic cavity. This type of ALL remains challenging even after the removal of the native liver and poses a problem for OLT. Furthermore, it is uncontemplated clinically and difficult to diagnose non-surgically (9).

Effective hepatic venous drainage is particularly important in OLTs, and wider anastomosis is instrumental. To our knowledge, this is the first report of abnormal outflow of the liver, which differs from Budd–Chiari syndrome, in which obstruction to hepatic venous drainage occurs and causes ascites, edema, and hepatomegaly (10). Budd–Chiari syndrome may be due to obstruction without thrombosis. Conversely, for abnormal outflow, obstruction does not occur before OLT and can occur due to incorrect reconstruction of the liver outflow upon OLT. The reason for the two outflow orifices of the rat liver is unknown. Preoperative screening for these abnormalities in small animals is also important in real life. In reality, almost all researchers or microsurgeons directly perform liver transplantation on small animals such as mice or rats. These abnormalities are discovered unexpectedly. If possible, the recipients such as the mouse or rat, should be screened via ultrasound before operation, which is so convenient and cheap. Notably, we did not notice any other concurrent anatomic abnormalities in rats with accessory lobe/multiple outflows. To our knowledge, this is the first study to assess rare abnormalities related to OLT.

## Conclusion

Abnormalities of the host liver have a negative impact on OLT, suggesting that pretransplant detection of anatomical abnormalities is useful for clinical practice.

## Data Availability

The original contributions presented in the study are included in the article/Supplementary material, further inquiries can be directed to the corresponding authors.
